# Disentangling the influence of salience and familiarity on infant word learning: methodological advances

**DOI:** 10.3389/fpsyg.2013.00175

**Published:** 2013-04-17

**Authors:** Heather Bortfeld, Katie Shaw, Nicole Depowski

**Affiliations:** ^1^Department of Psychology, University of ConnecticutStorrs, CT, USA; ^2^Haskins LaboratoriesNew Haven, CT, USA

**Keywords:** near-infrared spectroscopy (NIRS), MMN (mismatch negativity), MMR (mismatch response), acoustic salience, acoustic familiarity, infant speech perception, repetition suppression effect

## Abstract

The initial stages of language learning involve a critical interaction between infants' environmental experience and their developing brains. The past several decades of research have produced important behavioral evidence of the many factors influencing this process, both on the part of the child and on the part of the environment that the child is in. The application of neurophysiological techniques to the study of early development has been augmenting these findings at a rapid pace. While the result is an accrual of data bridging the gap between brain and behavior, much work remains to make the link between behavioral evidence of infants' emerging sensitivities and neurophysiological evidence of changes in how their brains process information. Here we review the background behavioral data on how salience and familiarity in the auditory signal shape initial language learning. We follow this with a summary of more recent evidence of changes in infants' brain activity in response to specific aspects of speech. Our goal is to examine language learning through the lens of brain/environment interactions, ultimately focusing on changes in cortical processing of speech across the first year of life. We will ground our examination of recent brain data in the two auditory features initially outlined: salience and familiarity. Our own and others' findings on the influence of these two features reveal that they are key parameters in infants' emerging recognition of structure in the speech signal. Importantly, the evidence we review makes the critical link between behavioral and brain data. We discuss the importance of future work that makes this bridge as a means of moving the study of language development solidly into the domain of brain science.

## Salience and familiarity as guides to segmenting the speech signal

Here we synthesize recent findings on changes in infants' brain activity in response to specific aspects of speech. In particular, we focus on two aspects of the speech signal that have been shown to influence infants' emerging sensitivities: acoustic salience and the familiarity of auditory form. These are not easy things to disentangle. Whether or not this is, indeed, possible, recent findings on the influence of these features on infant auditory processing reveal that they are both fundamental to infants finding structure in the speech signal. Importantly, the evidence we review makes the critical link between behavioral and brain data, and hints at a resolution to the debate about whether familiarity and salience are distinct acoustic features or one and the same. At present, we define acoustic salience (or salience) as a construct that is based on factors external to the language learner; salience can be conceived of as those physical characteristics inherent to the stimulus itself that make it salient. We consider this kind of salience distinct from preferences based on prior exposure or experience. Prior experience is what establishes familiarity of auditory form (or familiarity). Familiarity is what the infant brings to the perceptual process. Based on their experience with language and the environment, infants should develop the initial basis for mental representations (which are not necessarily conscious) of strings of sounds. The emergence of a mental lexicon no doubt influences how acoustic stimuli are subsequently processed (e.g., eventually as words). Here we review key behavioral findings demonstrating how these two features interact to influence infant speech processing. We then link these findings to more recent brain-based measures of infant processing. Finally, we review a methodological advance in use in our own lab, near-infrared spectroscopy (NIRS), including some initial data obtained using NIRS, that point to the utility of this method for teasing apart the relative influences of acoustic salience and familiarity in early language learning.

## Behavioral evidence of the influence of salience and familiarity

One of the most common examples of salience is inherent in the acoustic features that characterize infant directed speech (henceforth, IDS). Speech directed to infants generally consists of patterns of exaggerated pitch and rhythm, causing infants to prefer it to adult-directed speech (ADS) (Fernald, 1985; Fernald and Kuhl, 1987; Cooper and Aslin, 1990). This preference on the part of infants is well established. Early tests of this typically relied on a visual-fixation procedure to establish whether infants preferred to listen to infant-directed over ADS. For example, in a test of newborns and 12-month-olds (Cooper and Aslin, 1990), results indicated that both the newborns and the 12-month-olds demonstrated increased visual fixation during IDS trials, suggesting a preference for IDS over ADS. More recently, Thiessen et al. (2005) sought to determine whether the preference for IDS over ADS serves an infant's learning needs, particularly in the language domain. These researchers also used a familiarization procedure and found that 6-to 8-month-old infants were better able to segment statistically instantiated items out of a speech stream when they had originally been presented to the infants in IDS. This suggests that, not only do infants prefer IDS, but that the salient characteristics of this form of speech help them recognize individual items within running speech. This is so even before infants are able to exhibit stable language production behaviors themselves.

Despite infants' general preference for IDS, their preference for specific affective content within this form is not constant and has been demonstrated to shift during the first year. For example, 3-month-olds were found to prefer IDS that was pre-rated as soothing or comforting, 6-month-olds preferred IDS pre-rated as approving, and 9-month-olds preferred “directive affective” speech, a type of speech indicating that the infant should behave in a certain manner (Kitamura and Lam, 2009). This developmental shift in affective preference suggests that infants and caretakers influence one another over the course of the infant's first year in determining which type of speech will be most salient for the infant; caregivers are more likely to use a particular affective tone if the child is more likely to attend to it. Presumably, this facilitates language learning, since it promotes the mutual give-and-take that is the basis for communication in general.

If infant-directed speech is caregivers' way of making speech salient to infants, when does familiarity begin to play a role in their processing of the speech stream? As infants hear more and more IDS, it should become a familiar auditory form, both in terms of its general prosodic structure and, perhaps, in terms of the particular words it most frequently contains (e.g., an infant's own name). Indeed, familiarity can be derived from salience, as hypothesized by Snow (1972) based on her observation that the hallmarks of IDS include simple utterances and redundancy. While both of these characteristics increase the overall salience of this form of speech, such salience also provides a scaffold for word learning by highlighting particular forms within the acoustic stream. Eventually, those forms become familiar, thus facilitating additional structural learning. Support for this view comes from a study designed to investigate developmental differences in infants' preference for different aspects of IDS, in which 6-month old infants were found to prefer the repetitive structure, rather than their earlier preference for its prosodic elements (McRoberts et al., 2009). This shift in preference from the prosodic elements to the repetitive elements may be an indication of when infants transition from processing the general characteristics of the speech stream to recognizing components (i.e., words) within it. Such a view is consistent with findings showing that infants discriminate among words relatively early in life (Tincoff and Jusczyk, 1999; Bortfeld et al., 2005; Bergelson and Swingley, 2012).

## Interaction of salience and familiarity

Important evidence of the interaction of acoustic salience and familiarity in infants' speech processing comes from a study by Barker and Newman (2004). These researchers found that infants not only showed a preference for words spoken by their mother, but that they were able to attend to her voice in the presence of background noise that consisted of an unfamiliar female speaker. One implication of this finding is that, since infants can attend to their mothers' voices even in the presence of noise, they may be able to learn acoustic structure better from their mother (or from some other highly familiar individual) than when the words are being produced by an unfamiliar speaker. Regardless of who the speaker is, however, IDS contains important cues that appear to facilitate language learning. It also parallels patterns of speech between adults. When adults engage in conversation, the first instance of a word's utterance is typically more enunciated (clear) and longer than in subsequent references, suggesting that the speaker assumes a common ground between him or herself and the listener (Fowler and Housum, 1987). Repeated words are acoustically truncated in IDS as well, suggesting that such truncations may help draw infant attention to previously unsaid words (new information) in sentences that contain predominantly old information (Fisher and Tokura, 1995).

Additional research has revealed that this pattern is not stable when adults speak to infants, highlighting an interesting and important characteristic of language input. Bortfeld and Morgan (2010) investigated the given-new contract in infants by measuring the (several subsequent) repetitions of words produced by mothers in a single instance of speaking. Of note, such repetition is not something that adults would do when speaking with other adults; rather, the focal word is typically referred to with a pronoun after its initial one or two mentions. When speaking to infants, however, adults will repeat a word multiple times, providing an interesting pattern on which to perform acoustic analyses. Therefore, in their study, Bortfeld and Morgan (2010) did just this, finding that the second utterance of a word, when directed to infants was, indeed, truncated, and produced less emphatically, a finding that mirrors Fisher and Tokura's (1995) earlier results. However, when looking beyond the first two mentions of a word, it became clear that mothers revert to emphatically stressing that word all over again, followed again by de-emphasis. Given that adults will repeat a word to an infant sometimes six or eight or ten times, this points to a rhythmic production pattern that, while mirroring adults' speech, exaggerates it through repetition. Although the second (and subsequent) sets of repetitions may be less stressed and enunciated overall in comparison to the first, mothers nonetheless appear to revert to the same pattern of emphasis/de-emphasis. This is the case at least until they change the focus of their speech. Together, these findings provide support for the view that acoustic salience provides the foundation for familiarity. And familiarity, often in concert with salience, facilitates language learning. Of course, a form may become more salient to an infant as its familiarity increases (e.g., by taking on semantic meaning). But if we constrain our characterization of salience to acoustic salience, the directionality of influence implied here makes sense, and appears to hold for individual speech sounds as well. For example, a recent study (Narayan et al., 2010) challenges the long-standing view that infants can discriminate all functionally discriminable (i.e., categorically distinct) sounds. Instead, Narayan et al. (2010) observed a case in which acoustic salience (in the form of more versus less discriminability) interacts with an infant's environmental exposure. Their work suggests that differential discriminability is not entirely consistent with the all-to-some view of perceptual tuning patterns across the first year of life. Specifically, the researchers focused on Filipino, a language in which there is a subtle difference in nasalization between /na/ and /ηa/ that does not exist in English; on the other hand, the contrast between /ma/ and /na/ exists in both languages and is much more salient to the listener. English-exposed infants were shown to discriminate /ma/ from /na/ at both 6-to-8 and 10-to-12 months of age, but they were not able to discriminate non-native and less acoustically salient /na/ vs. /ηa/ contrast at either of these ages. Even very young (e.g., 4-to-5 months of age) English-exposed infants showed discrimination of only the former (/ma/ vs. /na/) and not the latter contrast (/na/ vs. /ηa/). Notably, Filipino-exposed infants showed discrimination of their native [na]-[ηa] between 10- and 12-months, but not between 6- and 8-months. This pattern of findings suggests that experience is necessary to establish long-term discrimination of two very similar speech sounds (e.g., /na/ and /ηa/), while acoustic salience enhances perception of very different sounds (e.g., /ma/ and /na/), providing a more nuanced view of how early perceptual reorganization unfolds.

Of course, different languages are characterized by differences well beyond the phoneme level. For example, it has been hypothesized that infants from different language backgrounds develop preferences for their particular (native-language) stress pattern early in life. To test this hypothesis, Hohle et al. (2009) conducted four experiments with German- and French-exposed infants at both 4- and 6-months. These languages have a notable contrast in stress, with German showing a strong trochaic (strong-weak) pattern that French does not have. At 4-months, German-exposed infants showed no preference for stress pattern; however, at 6-months, they began to show a preference for the trochaic pattern. On the other hand, French-exposed infants did not show a preference for one or the other pattern at 6-months, but were able to discriminate between the two. As with the phoneme discrimination findings, these results suggest that infants' sensitivities are shaped both by their environmental exposure and the absolute salience of the acoustic characteristic in question. Where trochees are quite salient in German, French's syllable timing rendered the trochaic form less salient to French-exposed infants.

Although acoustic salience is a useful tool for infants who are initially learning language, it can present problems as well. For example, if infants pay attention to their world based only on the physical salience of an object (auditory or otherwise), they may be missing other important aspects of the environment. When the item in question is a visually presented object, this can also affect the likelihood that infants will learn about other objects. In a clever study, Pruden et al. (2006) exposed 10-month-olds to a salient object (e.g., a glittery wand) and a less salient object (e.g., a beige bottle opener), while pairing each with a unique and novel auditory label. Despite being asked to identify the non-salient object, infants tended to look more at the salient object. Clearly, infants' tendency to attend to the salient things in the world around them doesn't always facilitate language learning.

Behavioral research has revealed several other sensitivities that infants bring to the learning environment. Consistent with the trochaic bias observed by Hohle et al. (2009) in German- but not French-exposed 6-month-olds, different languages have different units of segmentation. French tends toward the syllable, English and German use stress, and Japanese uses the *mora* (a subsyllabic unit) for segmentation (Cutler and Mehler, 1993). Indeed, earlier research demonstrated that infants exposed to each of these languages approach speech segmentation differently. A French-exposed infant, upon hearing Japanese, will segment the speech stream based on syllables, when the *mora* would actually be more appropriate (Cutler and Mehler, 1993). Although the means of segmentation are different in each language, the methods are similar: infants appear to recognize ambient rhythmic patterns early on, and use these patterns to segment the speech stream, thereby developing more precise awareness of the sounds within those segments. Again, although the familiar structures differ across languages, the general pattern is for those aspects of the environment which are the most salient to infants to become the most familiar (or at least to become familiar faster).

Infants are also sensitive to statistical regularities in their environment, using them as a guide to structure (e.g., Saffran et al., 1996). Beyond basic sensitivities, infants can then map these regularities to simple visual objects, demonstrating the first step in making label-object associations. For example, in a recent study (e.g., Shukla et al., 2011), infants were presented with a continuous speech stream and were able to recognize relationships between co-occurring segments (e.g., statistical “words”) and objects in the environment, but only if there was a high probability for co-occurring syllables (see also Graf Estes et al., 2007; Hay et al., 2011). This ability was extinguished when these statistically co-occurring segments crossed prosodic boundaries. These results are consistent with other work showing that prosody is a salient cue to infants by 6-months of age (see Kitamura and Lam, 2009; McRoberts et al., 2009) and that it interacts with their emerging sensitivity to structure. The fact that infants can map newly recognized structure onto simple visual objects (or at least associate them) demonstrates that the interaction of perceptual salience and familiarity forms the basis for active learning about relationships in the environment.

This happens at a more granular level as well. For example, the statistical likelihood of a sound string like “bref” is relatively high in English; one like “febr” is quite low. Mattys and Jusczyk (2001) observed that American English-exposed 9-month-olds segmented words as a result of the likelihood of the phoneme sequences in their language of exposure (in this case, American English). In other words, their familiarity with their own language's phonotactic structure actively influenced what infants found perceptually salient by the end of the first year. Graf Estes et al. (2011) expanded on this work by using a looking-while-listening paradigm with 18-month-olds. In this, infants were first presented with two object labels that were paired with novel objects. These labels were either legal (contained sound sequences that frequently occur in English) or illegal (contained sound sequences that never occur in English). At test, infants looked at the correct object when presented with the legal label; they did not look at the correct object when presented with the illegal label. These results demonstrate that phonotactic sensitivities have the power to shape learning.

In earlier work (Bortfeld et al., 2005), my colleagues and I demonstrated that infants can use existing words to scaffold their learning of new words. Specifically, we found that 6-month-olds can learn a new word if they had been familiarized with it while it was consistently preceded by either their own name or some other highly familiar name (e.g., mommy/mamma, depending on which term the mother used to refer to herself). Names for important individuals (e.g., oneself, one's primary caregiver) are highly frequent and thus become very familiar. This study shows that such familiarity can serve as a tool for subsequent segmentation of the speech stream, thereby facilitating progressive language learning. In this case, it is unclear which comes first, salience or familiarity. Presumably the semantic meaning associated with the familiar sound string is what brings the salience to the word, an important caveat to the argument laid out earlier about salience leading familiarity. And familiarity can sometimes undermine learning. In a clever study, Houston and Jusczyk (2000) familiarized infants with words produced by one speaker and then tested whether they could generalize their learning to unfamiliar speakers and to unfamiliar contexts (an ability that would reveal a more abstract form of representation). Results suggested that such abstraction did not happen, at least initially. Of course, speaker-specific representation of words is not a very functional way to learn language; fortunately for everybody, infants' retention of indexical information about individual speakers attenuates by about 10.5-months of age.

We have reviewed just a smattering of the behavioral evidence supporting the role of salience and familiarity in language development. Whether conceptualized as one or two identifiable characteristics of acoustic form, many questions remain. In particular, it is not always clear whether familiarity and/or salience act in a top-down or bottom-up manner. Salience may enter the system, at least initially, in a bottom-up manner (e.g., from the environment; from biologically established biases toward the environment) and thereby shape developing representations. Then again, it may not.

In a final example of the complex interaction between new and learned information in the process of language learning, Mersad and Nazzi (2012) used statistical learning in combination with familiar form. In a tweak of the usual approach to testing statistical learning, these researchers used non-uniform length novel words instead of the standard uniform-length novel “words” from the audio stream. Eight-month-olds were hindered in their ability to segment these non-uniform length novel words when presented with no other cues. However, they could segment the non-uniform length novel words when the words were preceded with a familiar word (*maman*, French for mom). In other words, what had become salient (“*maman*”) through initial familiarization provided infants with top-down guidance for parsing a complex (bottom-up) signal. This is just another demonstration of the degree to which top-down and bottom-up processes are interacting in complicated ways—from an early age and all along—to influence language processing. Ultimately, these data highlight the challenge inherent in characterizing which came first in any form of infant perception, salience, or familiarity.

## A way forward? brain activity distinguishes the influence of salience and familiarity

Thus far, we have focused exclusively on studies in which behavioral measures were used to investigate how infants process speech. Indeed, infants' overt gaze and sucking behaviors have provided us with important insights into their perceptual experiences, and behavioral measures are foundational in our understanding of how humans begin learning language. However, limitations to the interpretations that can be made based on these measures remain. For example, it is often difficult to tell with certainty what exactly both the looking time and the looks themselves signify (for a cogent review of the issues, see Aslin, 2007). Increasingly, researchers are turning to the growing array of neurophysiological methods that can be used with infants to better understand what those looks mean. Neurophysiological techniques have aided our ability to assess and measure language development through the first year of life and beyond. Although some are still gaining ground in developmental studies (e.g., NIRS), other techniques [e.g., electroencephalography (EEG)] form the basis for our understanding of both the timing and neural correlates underlying language milestones. The continued integration of behavioral methods with one or more of these techniques holds great promise for the advancement of language learning research, in particular, and developmental research, in general.

## EEGs and event-related potentials

One well-established technique for use with infant populations is EEG, a non-invasive tool with excellent temporal resolution and mild to moderate spatial resolution (for a review, see Fava et al., 2011). The application of this technique to research with preverbal infants has allowed researchers to pinpoint, in tens of milliseconds (ms), when sensory processing is occurring. It also provides information about different processing stages. The non-invasive nature of EEG makes it a relatively safe procedure to use when studying infants, and a multitude of event-related potentials (ERPs) can be assessed, even in neonates (Korotchikova et al., 2009). In addition, EEG can provide data without requiring a behavioral response. This is especially valuable when testing very young infants, who often are unable to produce reliable behavior in response to perceptual stimuli, and when the goal is to determine when an infant notices a stimulus change.

The workhorse of ERP research, the Mismatch Negativity (MMN) component, is one that has been widely used with both infants and adults. The MMN is measured in the 150–250 ms window of time, post-stimulus onset. When presented with a sequential list of identical exemplars, the adult MMN has been found to have higher amplitude for deviant stimuli (e.g., an oddball) (Naatanen, 1995). One of the hallmarks of the MMN is that it is relatively impervious to conscious modulations in attention and thus can be found even when a person is not focusing on the stimuli (Luck, 2005). In adults, the MMN has been observed in response to auditory stimuli even while the individual is engaging in an unassociated cognitive task, such as reading. This has led to the view that the MMN reflects processing that is pre-attentive and passive (Alho et al., 1992), making it an ideal candidate for use with infants. There has been considerable debate over whether the early time window of the MMN and the factors shown to modulate it are the result of bottom-up perceptual processing alone, particularly in low-level acoustic change detection tasks (Kenemans and Kahkonen, 2011). Several studies have demonstrated a dynamic interaction between salience (bottom-up effects) and familiarity (top-down effects) in MMN amplitudes (for review, see Garrido et al., 2009). The possibility that the measure may get at the interplay between features such as salience and familiarity in early processing underlies its promise for additional infant research on precisely this issue. Thus far, however, much of the infant-specific research has focused on stimulus familiarity as the basis for the change in voltage amplitudes.

In an influential early study, behavioral techniques revealed that infants prefer to listen to their mother's voice relative to that of a stranger (DeCasper and Fifer, 1980). Indeed, and as noted earlier, they can even distinguish their mother's voice in the presence of noise (Barker and Newman, 2004). Beauchemin et al. (2011) sought to better understand the basis for this preference by using the mismatch response (or MMR), a developmental precursor of the mismatch negativity response seen in adults, and source analyses (for cortical localization) during infants' processing of familiar voices. The researchers tested neonates between the ages of 8- and 27-h while they were exposed to a concatenated stream of the French vowel “a” (as in “allo,” the French pronunciation of “hello”) produced by an unfamiliar female speaker. Two types of auditory oddballs were inserted into the speech stream fifteen percent of the time, either a different unfamiliar female producing “a” or the infant's own mother producing “a.” They found that when presented with the mother's voice as an oddball stimulus, MMR amplitudes were significantly greater than MMR amplitudes measured when the second stranger's voice was an oddball stimulus. This finding suggests that familiarity (in this case, with the mother's voice) is in play from birth, thereby influencing auditory processing beyond simple acoustic change detection.

In addition to analyzing the MMR Beauchemin et al. (2011) also conducted source analyses to better gauge not only when but where these modulations were occurring neurophysiologically. They found that the mother's voice activated the left posterior temporal lobe throughout the first 300 ms of exposure, while the stranger's voice activated the right temporal lobe (~100 ms), followed by a switch to the left temporal areas (200 ms), and then a reversion back to the right temporal lobe (~300 ms). The authors interpret the lateralized response to the mother's voice as demonstrating earlier recognition of the stimulus as being a language component, as well as evidence that the tuning of voice specific recognition in the brain occurs within the first 24 h after birth. Of course, there remains some skepticism about the accuracy of EEG-based source localization (see Plummer et al., 2008), so these results should be interpreted with caution.

As we have observed based on our review of behavioral data, multiple forms of familiarity may influence infant language learning, well beyond the mother's voice. Familiarity, and thus preference, for a number of aspects of the signal may help the infant begin to segment fluent speech and to learn new words. For example, focusing on sensitivity to stress patterns, Weber et al. (2004) compared 4- and 5-month-old infants German-exposed infants with native German speaking adults. Specifically, they looked at participants' MMR to consonant-vowel-consonant-vowel (CVCV) sequences produced with either trochaic stress (e.g., stress placed on the initial syllable and typical of the German language) or iambic stress (e.g., stress placed on the second syllable and atypical in German). Half of the participants experienced the trochaically stressed words as “standards” and the iambically stressed words as the MMR-dependent “deviants.” The reverse was true for the other half of the participants. For the adults, an MMR occurred whether the deviant was either a trochaic or iambic string, suggesting that adults were sensitive to both stress patterns when they were novel relative to the ongoing auditory stream. However, for infants, an MMR was observed in the 5-month-olds for deviant trochaic stimuli only, while neither stress type provoked a significant MMR in the 4-month-olds. This suggests that between 4- and 5-months of age, infants become increasingly tuned to the most common stress patterns of their exposure language, though they have yet to reach adult-like discrimination abilities for unfamiliar stress patterns. This is consistent with the behavioral findings (e.g., Hohle et al., 2009), allowing us to infer that sensitivity to stress patterns are experience-dependent and emerge during the course of preverbal language exposure.

In-line with behavioral studies investigating the influence of familiarity on infant speech segmentation (e.g., Bortfeld et al., 2005), ERP studies have also demonstrated a privileged role for familiar words presented in continuous speech. Kooijman et al. (2005) familiarized 10-month-olds to bisyllabic words, presented in isolation, following the stress pattern of their native language (Dutch) and then presented in sentences at test. During the familiarization phase, enhanced ERP responses were found during word presentation in the frontal, fronto-central, and fronto-temporal regions while at test they were more left lateralized, suggesting different underlying neural processing mechanisms. Importantly, these effects were found prior to word offset, suggesting that infants were recognizing the newly familiarized words based on the first syllable and stress pattern. These findings demonstrate the neural underpinnings involved in speech stream segmentation and provide further evidence of word familiarity influencing said segmentation. In a follow-up study, Junge et al. (2012) further examined the relationship between word familiarization and vocabulary development by longitudinally assessing ERPs at 10-months-old as being predictive of vocabulary development at 12- and 24-months-old. They found that infants who demonstrated better segmentation abilities at 10-months of age also had higher vocabularies at 12- and 24-months-old, suggesting that rapid recognition of words is an integral part of language development and may be useful in understanding individual differences in vocabulary acquisition.

These results, while compelling evidence of the utility of the MMN in infant research, all serve as additional support for the importance of familiarity in infant processing and thus move us no closer to our goal of understanding the interplay between that and acoustic salience. Another common ERP component, the N400, may highlight a way forward. The N400 has been used extensively in language research in both infants and adults (de Haan et al., 2003). This component is characterized by a negative peak amplitude around 400 ms post-stimulus-onset, although the time window ranges from 250 to 500 ms. Higher N400 amplitudes have been found in adults for sentential semantic violations (e.g., *Bill is lactose intolerant therefore he drinks milk*), although violations within individual words have also resulted in higher amplitudes (Kutas and Federmeier, 2011). The N400 is also influenced by semantic priming in adults (Kutas and Hillyard, 1980), a response elicited by a level of processing typically unexpected in infant research.

However, in a recent study, Parise and Csibra (2012) investigated whether the N400 could be modulated in 9-month-olds by presenting a spoken referent that was inconsistent with a visually presented object. The researchers hypothesized that if an N400 was evident for a mismatch between the auditory and visual modalities, then it would represent infants' association of the heard label with a particular visual stimulus. They further reasoned that if an N400 was not found for a mismatch between object and label, then this would demonstrate that infants may be relying on temporal associations when pairing words with objects and *not* semantic representation. In the study, a mother or a stranger produced a familiar object label. Two seconds later, an occluder was removed, displaying an object. Results showed that when the object did not match the label as spoken by the mother, the N400 response was greater in amplitude, suggesting that infants processed the discrepancy at a semantic level. In contrast, the N400 was attenuated in both match and mismatch trials for the stranger's production of the object label, suggesting that the semantic representation was specific to the mother's voice, and that infants were not yet abstracting their representation across exemplars of the word. These results are consistent with other demonstrations of the important role of a consistent acoustic source (e.g., the mother) in infant language development. But they also hint at a way of getting at the dynamic interplay between familiarity and salience in early word learning: one could argue that the infants' initial semantic representations for the familiar objects were based in the salient acoustic form (e.g., the mother's voice). While it can still be argued that the mother's voice is salient precisely because of its familiarity, it should be clear that the addition of a semantic-level component to the infant ERP toolkit is an important step toward our ability to tease apart the relative influence of familiarity and salience.

In another study investigating the N400 in early language development, Friedrich and Friederici (2005a) compared response activation to phonotactically legal (pseudowords) and illegal words (nonsense words) in 12-month-olds, 19-month-olds, and adults paired with objects. Pseudowords followed the phonotactic rules of the participant's native language (German) while nonsense words violated phonotactic constraints. These researchers found strong evidence of an N400 effect in 19-month-olds for pseudowords over nonsense words when paired with an object, suggesting that prior knowledge of the phonotactic constraints of the native language influence which words can be used as object referents. In contrast, 12-month-olds did not show differences in N400 amplitude based on legality of the words, which the authors assert may reflect a lack of maturity in the N400 ERP. Overall, their study provides additional evidence that familiarity with phonotactic rules of the native language influence word processing and object referencing, particularly in the second year of life, a finding that is consistent with other findings from these researchers (Friedrich and Friederici, 2004, 2005b). Still others have observed enhanced ERPs for newly-learned words in 20-month-old infants, mirroring their response to previously known words in object-pairings, albeit at an earlier time-window (N200–N500; Mills et al., 2005).

The only clear examination of salience as it interacts with familiarity in infant speech processing comes from a study using both early and late time-course ERPs in combination. Specifically, Zangl and Mills (2007) investigated how familiar and unfamiliar words presented in IDS or ADS affected the N200–N400 time-window amplitude and the Nc component in 6- and 13-month-olds. The Nc component is a mid-latency, negative-going waveform characteristic of the fronto-central scalp regions (Richards, 2003). Importantly, it is considered an endogenous attentional component, reflecting top-down influences on attentional orienting and perceptual processing (Richards, 2003), and thus is relevant for understanding how previous experience may facilitate subsequent processing. The researchers found that 13-month-olds, but not 6-month-olds, showed enhanced N200–N400 amplitudes for familiar words presented in IDS over familiar words presented in ADS, but showed such no difference for unfamiliar words. Regardless of age, the Nc component was greater in amplitude for IDS over ADS, suggesting that infants increased attention to the speech stream as a result of the more salient speech register. Together, these findings suggest that exposure format (e.g., more or less salient speech type) and exposure form (e.g., word familiarity) interact in driving infant attention toward speech in the first year of life. More research along this line is sorely needed.

Clearly, EEG (and accompanying ERPs) is an established and important tool for assessing infant perception without requiring explicit behavior. Electrophysiological studies have provided a bridge to better understanding of the neural basis for a variety of behavioral findings. Source localization techniques notwithstanding, the limited spatial resolution of this particular methodology constrains the inferences that can be made about which areas of the brain are developing when, and what their role in early speech processing is. More recently, novel hemodynamic-based techniques (e.g., NIRS) have emerged for application with infant populations, as has the application of established hemodynamic-based techniques (e.g., fMRI) to infant populations. To better understand how neural development facilitates the integration of salience and familiarity in the service of language learning, it is worth examining data from this domain of infant research as well.

## Hemodynamic-based measures

In an influential early developmental imaging study, Dehaene-Lambertz et al. (2002) tracked changes in cerebral blood flow in 2-to-3-month-old infants using fMRI while the infants were exposed to samples of forward and backward speech in their native French. Infants were tightly swaddled prior to being placed in the core, so as to restrain their movement. They were presented with recordings of a woman reading passages from a children's book. The passages were either presented normally (e.g., forward speech) or the recordings were time-reversed (e.g., backward speech). The researchers hypothesized that brain regions associated with segmental and suprasegmental speech processing would be more highly active during exposure to typical, forward speech. In contrast, the backward speech condition should violate phonological properties of the infants' native language, and thus, activation in the brain regions sensitive to speech structure should be less active in response to it. Results revealed that, indeed, brain regions were differentially activated as a result of speech condition. During exposure to forward speech, infants' left angular gyrus and left precuneus were significantly activated, suggesting that infants were not only recognizing the familiar acoustic structure during the forward segments (see Démonet et al., 1992 for adult comparison of left angular gyrus), but also engaging in early memory retrieval (see Cavanna and Trimble, 2006 for adult comparison of left precuneus). Of course, because the infants were swaddled, they fell asleep during much of the testing in this study. The researchers coded for sleep state based on their observations of infants' faces during testing. Although many of the results were not influenced by sleep state, it is worth noting that there was some variability in the data based on it that will require additional research to better understand.

Functional studies have likewise provided evidence of infants' sensitivity to a familiar speaker (e.g., Dehaene-Lambertz et al., 2010). In this study, the researchers used fMRI to investigate the neural correlates of speech perception in 2-to-3-month-old infants, specifically comparing speech produced by their own mother to that produced by a stranger, as well as speech versus music. Results revealed that, even by 2-months of age, infants showed left-lateralized processing of speech relative to music, and that this lateralization of activation was modulated by whether the voice was familiar or not. During exposure to their mothers' voice, infants' left posterior temporal region was more highly activate than during exposure to a stranger's voice, suggesting that low-level acoustic familiarity enhances speech-specific processing. These results are consistent with the behavioral findings reviewed earlier from Barker and Newman (2004), as well as recent ERP results from Parise and Csibra (2012), showing an interaction of voice familiarity and semantic representation.

The feasibility of using functional magnetic resonance imaging and other motion sensitive techniques with very young populations is necessarily limited. While fMRI has excellent spatial resolution, it is generally quite noisy and also susceptible to motion artifacts. Researchers have to adjust study designs to account for the challenges of working with infant participants when planning and conducting studies. However, NIRS is a more infant-friendly hemodynamic-based measurement tool; it is non-invasive, less vulnerable to motion artifacts, and safe to use even with newborns (Sakatani et al., 1999; see Aslin, 2012 for a comprehensive review of this technique and its application in infant research).

Near-infrared spectroscopy is providing important insight into the dynamic interaction of a number of factors on how preverbal infants process speech and how this changes in developmental time. For example, using NIRS, Homae et al. (2006), (2007) investigated developmental changes in cortical activation specific to prosody in 3- and 10-month-old infants. They sought to determine when the right lateralization that is typical of prosodic processing in adults (Baum and Pell, 1999) is evident in infants. In their study, infants were presented with both normal and flattened speech, in which the flattened speech was void of pitch contours. They found that 3-month-olds displayed bilateral activation in the temporoparietal, temporal, and frontal regions for both speech types and enhanced activation in the right temporoparietal regions for natural speech (Homae et al., 2006). These findings suggest that even by 3-months of age, infants are sensitive to the prosodic information available in the speech signal. In addition, a follow-up study with 10-month-olds (Homae et al., 2007) using the same methodology, found greater activation in the right temporoparietal and temporal regions for prosodically flattened speech in comparison to natural speech, mirroring adult patterns. The authors assert that the differences between their two findings demonstrate a developmental shift in pitch processing mechanisms as a result of greater experience with the prosody of the child's native language.

## Combining brain and behavior: repetition suppression

To assess the cortical changes that underlie advances in language in the first and second years of life, my colleagues and I have been using another hemodynamic-based measurement technique, NIRS (Bortfeld et al., 2007, 2009). Specific to the current focus on how the infant brain is shaped by salience and familiarity, we have been using NIRS with a well-established behavioral protocol. The results, which we will review here, are promising.

As should be apparent from this review, a common tool for studying infants' sensitivity to stimuli (or specific characteristics of stimuli) is to establish response habituation based on looking times. This is something that can likewise be used to study brain responses (e.g., Turk-Browne et al., 2008). In the fMRI literature, habituation to stimulus characteristics is observed in the form of repetition suppression (Grill-Spector et al., 2006), whereby prior exposure to stimuli (or stimulus attributes) decreases the level of activation elicited during subsequent exposure to identical stimuli. Although the underlying neuronal mechanisms remain unclear (for review and discussion, see Henson, 2003; Henson and Rugg, 2003), repetition suppression has been interpreted as the fMRI analog of neuronal response suppression observed using single cell recording (Desimone, 1996). This reduction in brain activation with repeated exposure presents an ideal scenario for establishing whether infants' brains show a decrease in hemodynamic activation concomitant with a decrease in looking (i.e., over the course of habituation), a demonstration of increased familiarity.

When repetition effects are present in a brain region in human adults, they indicate that the particular region (showing a reduction in activation) is supporting the representation of the stimulus, and variants of the paradigm have been used to monitor the abstractness of a particular representation (Grill-Spector and Malach, 2001; Naccache and Dehaene, 2001). For example, the left inferior frontal region appears to be quite sensitive to sentence repetition, suggesting that it is part of the network supporting early verbal working memory, at least in adults (Dehaene-Lambertz et al., 2006a). In newborns, the repetition of a syllable every 600 ms produced a decrease in ERP amplitudes (Dehaene-Lambertz and Dehaene, 1994; Dehaene-Lambertz and Peña, 2001) and in a more recent study (Dehaene-Lambertz et al., 2010), repetition suppression was observed in 2-month-olds exposed to repetition of the same sentence at 4 s intervals. In infants, this repetition suppression was observed in the left superior temporal gyrus, extending toward the superior temporal sulcus and the middle temporal gyrus. However, a slow event-related paradigm where a single sentence was repeated at much longer (e.g., 14 s) intervals did not produce any repetition suppression (Dehaene-Lambertz et al., 2006b), which may point to the limits of the early verbal working memory window. Of course, the absence of a repetition suppression effect in this case could have been related to any number of factors (e.g., unique characteristics of the BOLD response in infants, complexity of the sentence, or, indeed, the extended time-lag erasing the echoic buffer of the temporal regions).

**Figure 1:**
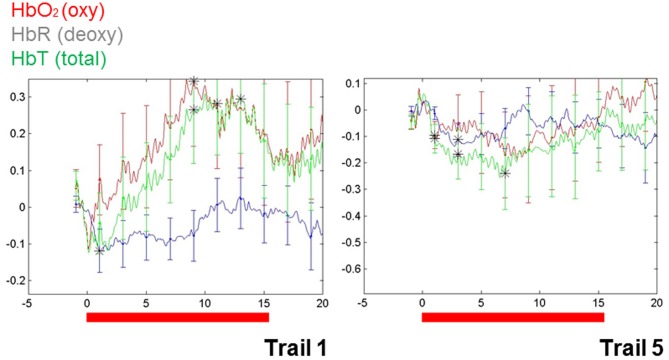
**Changes in blood flow in a single 9-month-old infant during the first and the final (fifth) series of repetitions of a single, monosyllabic word.** The Y-axis is relative changes in concentration (micromolar) and the X-axis is time. Area of recording is left superior temporal gyrus, with the optode centered over T3 (of the 10–20 system).

These findings do, however, highlight a way forward. Importantly, repetition suppression was observed with immediate repetition in these infants, providing a methodological vehicle for clarifying characteristics of auditory representation in infants. More recently, repetition suppression has been observed in infant blood flow data collected using NIRS (e.g., Nakano et al., 2009). In our own work, the utility of repetition suppression has been tested using a mixed stimulus presentation combining aspects of both event-related and block designs. In this approach, we presented infants with individual stimuli repeatedly and with relatively short ISIs (e.g., 3 s). Test blocks were intermixed with control blocks (e.g., sets of comparable but variable stimuli). Initial data from a single (9-month-old) infant (see Figure 1) show a repetition suppression effect for the auditory repetitions of an individual word. That is, as a single word was repeated, the activation pattern over the left temporal region decreased with each subsequent repetition (e.g., as seen in the overall hemodynamic response reduction from the first 15 s of word repetition in Trial 1 to the final repetition in Trial 5). Furthermore, novel words that were matched for stress pattern, syllable count, and overall length in control blocks elicited a relatively sustained hemodynamic response in the same cortical location, highlighting the selectivity of the effect.

While these data speak to the brain's changing response with increasing familiarity, one can imagine more complex designs that would work toward differentiating response to both familiarity and salience in the same brain. And really, a robust hemodynamic response to a novel stimulus is an indicator of salience, particularly when compared to the same region's response after multiple repetitions of exposure. One approach using NIRS alone to resolve the salience/familiarity puzzle would be to introduce variations of form (e.g., changes in speaker; changes in pitch) to monitor a “release” from repetition suppression. Such a result would reveal in real time the brain's response to salient changes in the environment and, thus, to salience.

Together with the MMR approach outlined earlier, which pinpoints low-level responses to salient characteristics of the signal, the repetition suppression effect in hemodynamic based measures highlights a way forward. Importantly, NIRS very often reveals such effects on a trial-by-trial basis and in a single subject, something EEG data would be hard-pressed to do. Regardless, the stimulus selectivity of each measure makes them both useful tools for assessing early language processing. In particular, the repetition suppression effect can reveal the point at which a stimulus becomes familiar (or at least begins transitioning toward that state) and (presumably) what changes in that stimulus make it salient again. If familiarity is the basis for the development of representations of words, then a child's failure to show a typical repetition suppression effect may highlight a corresponding failure to encode relevant features of that word (e.g., the temporal order of individual sounds within it; its prosodic form). Such an effect can thus be exploited in a clinical setting as well, potentially providing important diagnostic information into the degree to which a child is (or is not) developing robust lexical representations. It could also be used to establish which feature changes in a stimulus make it salient again. All of these are possibilities that at least hint at a way forward in disentangling influences of salience and familiarity is early learning.

Ultimately, large scale, within-subject data collection will establish the utility of both the MMN and repetition suppression effects in research on infant perceptual processing. For example, blood flow measures collected in a canonical repetition suppression task and electrophysiological measures collected during a canonical mismatched negativity task could be related to subsequent language outcome on a child-by-child basis. For now, we can at least appreciate the complimentary nature of these neurophysiological techniques, both with one another and with the long history of careful behavioral testing that is critical to understanding infant perceptual development. These tools may yet reveal how salience begets familiarity (and vice versa).

Certainly there are limitations in the application of NIRS in infant research, and these should be taken into account when designing and conducting experiments (see Aslin, 2012, for review). Although NIRS is similar to fMRI in that it relies on measuring hemodynamic responses, it is severely more limited in its ability to gauge response from deeper brain structures (e.g., below the level of the cortex). It is optimally suited for examining structures near the cortical surface, ideally with probe design controlling for scalp-surface distance (Beauchamp et al., 2011). Additionally, because NIRS relies on changes in blood oxygenation levels, it has poor temporal resolution. Although the sampling rate for NIRS can far surpass that of fMRI, due to the inherent constraints on blood flow timing it is, for practical purposes, on par with that of fMRI. Finally, best practices for the application of NIRS research include attention to the development of approaches to signal processing and statistical analysis, as well as to probe design, all of which are needed to facilitate replication and cross-study validation of results. Nevertheless, the puzzle of how the developing brain integrates and assigns meaning to auditory information on its way to language is an important one to keep struggling with. The techniques reviewed here will no doubt contribute to our finding the solution.

### Conflict of interest statement

The authors declare that the research was conducted in the absence of any commercial or financial relationships that could be construed as a potential conflict of interest.
